# On-Line Composition Analysis of Complex Hydrocarbon Streams by Time-Resolved Fourier Transform Infrared Spectroscopy and Ion–Molecule Reaction Mass Spectrometry

**DOI:** 10.1021/acs.analchem.1c01929

**Published:** 2021-09-22

**Authors:** Christopher Sauer, Anders Lorén, Andreas Schaefer, Per-Anders Carlsson

**Affiliations:** † Department of Chemistry and Chemical Engineering, Chalmers University of Technology, SE-412 96 Gothenburg, Sweden; ‡ Department of Chemistry and Materials, RISE Research Institutes of Sweden, SE-501 15 Borås, Sweden

## Abstract

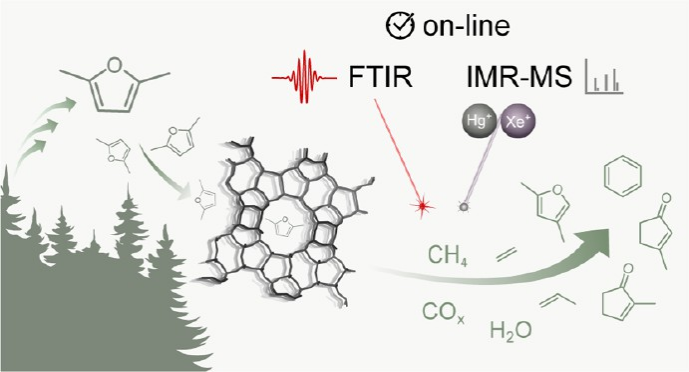

On-line composition analysis of complex hydrocarbon mixtures is highly desirable to determine the composition of process streams and to study chemical reactions in heterogeneous catalysis. Here, we show how the combination of time-resolved Fourier transform infrared spectroscopy and ion–molecule-reaction mass spectrometry (IMR-MS) can be used for compositional analysis of processed plant biomass streams. The method is based on the biomass-derived model compound 2,5-dimethylfuran and its potential catalytic conversion to valuable green aromatics, for example, benzene, toluene, and xylenes (BTX) over zeolite β. Numerous conversion products can be determined and quantified simultaneously in a temporal resolution of 4 min^–1^ without separation of individual compounds. The realization of this method enables us to study activity, selectivity, and changes in composition under transient reaction conditions. For example, increasing isomerization of 2,5-dimethylfuran to 2,4-dimethylfuran, 2-methyl-2-cyclopenten-1-one, and 2-methyl-2-cyclopenten-1-one is observed as the catalyst is exposed to the reactant, while BTX and olefin formation is decreasing.

## Introduction

Speciation and quantification of hydrocarbons (HCs) present in complex gas mixtures are important for research and development within many fields. Analysis of chemical process streams including plant biomass streams, atmospheric^1^ and environmental trace gas analysis,^2^ and combustion exhausts^3^ represents a few applications. The instrumentation for monitoring said processes^4,5^ is necessary for further development of chemical technology for feedstock valorization, exhaust aftertreatment, and monitoring anthropogenic^6^ and natural processes occurring in the environment.^7^ With the growing societal needs for the design of sustainable chemical products and processes utilizing renewable carbon feedstocks, the catalytic fast pyrolysis (CFP) of plant biomass presents an important area for development.^8^ From cellulosic biomass, various furans can be obtained.^9^ Both furan and 2,5-dimethylfuran (2,5-dmf) have been used as representative model compounds, demonstrating a route for upgrading biomass molecules into valuable aromatics such as benzene, toluene, and xylenes (BTX).^10−13^ For these conversions, mid-pore size (MFI and BEA) zeolites have been considered as suitable catalysts thanks to their surface properties hosting catalytically active sites and internal pore network offering shape selectivity toward aromatics.^14^

In the search for new chemical technologies for valorization of plant biomass, including further development of zeolite systems, it is of utmost importance that the rich product composition can be speciated and each species can be quantified. Even for CFP of model compounds, the HC product distribution is usually quite broad including several alkanes, alkenes, aromatics, and (partially) oxidized decomposition compounds.^10,15^ Furthermore, without useful methods for HC speciation and quantification, involved reactions and mechanistic pathways cannot be well-understood.

It is clear that improved analytical methods for continuous characterization of the product stream would pave the way for new knowledge that stimulates process development and catalyst research. Here, the conversion of 2,5-dimethylfuran to BTX over a zeolite catalyst will serve as the case around which we develop analytical methods for on-line analysis of gas phases rich in HCs.

Among the methods for the analysis of HC gas mixtures, one finds gas chromatographic separation in combination with, on the one hand, mass spectrometry (GC–MS) for identification and, on the other hand, flame ionization detection (GC–FID) for quantification. The separation step makes these methods superior in terms of standalone identification and reduced interference (cross-sensitivity) between different analytes even if separation of similar compounds is challenging and cannot always be guaranteed. Furthermore, light gases such as water and C_1_–C_3_ species might not be routinely analyzed.^16^ In the case of gas-phase reactions, conversion products are often collected in gas bags prior to analysis or condensed in liquid traps, which may complicate the analysis of original species.^11,17,18^ In response, on-line GC–MS has been developed. It was recently used to study the conversion of furan over zeolites, and several HC products were identified and quantified.^13,15,19^ Despite the on-line approach, the demonstrated time resolution was at best limited to several minutes. This is a general characteristic of separation-based methods that are of particular interest to circumvent as to enable studies of dynamic processes, for example, composition variations in process streams and catalyst activity, selectivity, and fast deactivation phenomena.

Fourier transform infrared (FTIR) spectroscopy and (quadrupole) mass spectrometry are both methods that can be conveniently used at time scales in the order of seconds. IR spectroscopy is widely used in research, such as for the quantitative analysis of HC mixtures,^20,21^ but also installed as a process analytical tool in the chemical industry.^5^ It relies on the interaction of light with chemical bonds in the probed molecule that is characterized by a permanent or induced dipole moment. The IR absorption is measured as a function of the wavenumber of the IR light giving rise to a spectrum containing absorption band characteristic of the molecule at hand. It is sufficiently sensitive and fast for many applications, but nonpolar molecules cannot be measured and for complex gas mixtures, overlapping IR bands are challenging to resolve. The latter may be overcome by tedious calibration and band deconvolution procedures. The principle of mass spectrometry is based on ionizing a sample, separation of the resulting ions according to their mass-to-charge ratio (*m*/*z*), and detection of the charged particles using an electron multiplier.

For ionization, several different techniques exist, resulting in a parent ion and (usually) a range of fragment ions depending on the analyte and ionization energy. Electron ionization mass spectrometry utilizes relatively high energy on impact, typically 70 eV, that creates many fragments of the parent ion, resulting in complex mass spectra with overlapping signals.

However, efforts have been made to develop universal gas analyzers that are based on proton-transfer reaction^22−24^ or ion–molecule reaction (IMR)^25,26^ principles. Both are categorized as chemical ionization techniques, where the latter one applies an ionizing gas as the primary ion source instead of protons or a reactant gas. This ionizing gas is itself first ionized in a separate chamber and then directed to the sample gas which it ionizes on impact, given that the ionization potential of the analyte ion is smaller than the one of the primary ion sources. In this work, Hg, Xe, or Kr are used as ionizing gases for IMR producing fragmentation patterns that are far less complex. Furthermore, the excellent sensitivity of MS complements the FTIR analysis when analyte concentrations are low and the combination of the two increases the likelihood of correct speciation in the case of spectral congestion. Both FTIR and MS are moreover well-suited to monitor in a solvent-free environment, which is often desirable for heterogeneous catalytic reactions.

Here, we present a methodology that combines on-line FTIR and IMR-MS for time-resolved quantification of a multitude of gaseous compounds originating from CFP of 2,5-dmf over a BEA zeolite catalyst in a laboratory reactor. The temporal resolution of 4 min^–1^ of the on-line method is a conservative choice that still provides much higher time resolution than that of separation-based analysis (typically 0.05 min^–1^ and up to 0.16 min^–1^).^10,15^ Specifically, we show how the method can be used to follow the formation of BTX, olefins, and other side products upon changing the feed composition and reactor temperature and thereby demonstrate its applicability for research studies on catalytic processes. We also report infrared spectra for 2,4-dimethylfuran and 2-methyl-2-cyclopentenone and mass spectra measured by Hg and Xe that can serve as references in future works. The pros and cons of the method are discussed and potential expansions of the method are envisaged.

## Experimental Section

### Target and Methodological Strategy

Reaching the goal of using a commercial FTIR spectrometer to quantify species in complex HC compositions dynamically requires an expansion of the commercial reference compound library. This library contains calibration files, that is, IR spectra and the corresponding compound concentrations, of a large number of organic and inorganic species sufficient for many analyses. However, several compounds of interest in this study are not part of the commercial compound library. To cover the entire range of relevant species, whereof many are not a priori known, catalytic experiments were carried out using a chemical flow reactor to produce these realistic species. The “heavy fraction” molecules in the product stream were collected with multisorbent tubes and identified with GC–MS. Complementarily, lighter species were identified through the analysis of infrared spectra for similar reaction systems in the open literature. Thereafter, calibration files for the missing compounds were created by systematically measuring the spectra of different concentrations of the pure compounds using high-purity chemicals and finally added to the reference compound library.

Furthermore, IMR-MS was used not only for monitoring non-IR-active molecules such as Ar and O_2_ but also to measure species whose concentrations are too low to be captured by infrared analysis and, for certain compounds, to validate the FTIR spectroscopic analysis.

### Analytical Instrumentation

For the on-line gas composition analysis, an FTIR analyzer (MKS MultiGas 2030) and a mass spectrometer (Airsense Compact, V&F) were used. The temperature and pressure in the FTIR cell were kept at 191 °C and atmospheric pressure, respectively. The inlet gas stream was conditioned to the same temperature also at atmospheric pressure. Spectra were collected between 500/600–4000 cm^–1^ with a resolution factor of 0.5 cm^–1^. The optical path length was 5.11 m. 16 spectra were averaged and recorded every 15 s resulting in a temporal resolution of 4 min^–1^. Background spectra were taken at 191 °C under a flow of pure argon. The collection of spectra was performed with the MKS MG2000 software suite v.10.2. and FTIR-library v. R3. The software includes a multivariate data analysis tool to make use of a large number of reference spectra. To create FTIR calibrations of additional compounds, the corresponding pure chemicals were used as listed below. To minimize cross-sensitivity and increase robustness, each calibration file utilizes the so-called primary analysis bands in the IR spectra, and using built-in functions, corrections for gas temperature and pressure variations are performed. From the measured absorption spectrum of a sample mixture, the concentration of each species is calculated by an algorithm based on classical least squares fitting of the primary analysis bands by the use of the multivariate data analysis tool.

The mass spectrometer for the on-line analysis was operated in the soft ionization mode using ion–molecule reactions. Hg (10.44 eV) and Xe (12.13 eV) were used as soft ionizers, offering different fragmentation patterns depending on the ionization potential. Software version V&F analyzer 1.4 was used.

GC–MS characterization was performed by sampling of the analytes from the product stream using adsorbent tubes (Tenax) which were analyzed via thermal desorption (Markes Thermal Desorber Unity2) into a GC–MS system (Agilent 7890A GC and Agilent 5975C MSD). The chromatographic separation was performed using a 60 m; 0.32 mm; 1.0 μm DB5-MS column ramped from 60 to 280 °C. The quadrupole mass spectrometer was operated using the EI-ionization and scanning mode between 29 *m*/*z* and 550 *m*/*z*. Spectra from an NIST GC/MS library were used to compare the collected spectra.

### Chemicals and Catalytic Material

Liquid compounds used for FTIR calibrations included 2,5-dimethylfuran (Sigma-Aldrich, ≥99%), 2,4-dimethylfuran (ABBlocks, ≥95%), 2-methyl-furan (Sigma-Aldrich, 99%), furan (Sigma-Aldrich, 99%), 2-methyl-2-cyclopenten-1-one (Merck/Sigma Aldrich, 98%), and 3-methyl-2-cyclopenten-1-one (Merck/Sigma Aldrich, 97%).

Zeolite β (Zeolyst, CP814C*, SiO_2_/Al_2_O_3_ = 38) was used as a catalytic material. A couple of monolith catalysts with ca. 160 mg zeolite β were prepared by dip-coating cordierite substrates (Corning, 400 cpsi, 188 channels, length = 15 mm, Ø = 13 mm) with a water-based slurry containing zeolite β powder and binder material (Ludox AS-40). Thereafter, the coated monoliths were dried at 200 °C and calcined at ca. 500 °C with a heat gun and the procedure was repeated until 200 mg of the dried mixture was attached to each substrate.

### Chemical Flow Reactor

The chemical flow reactor consists of a quartz tube surrounded by a metal coil, which is thermally insulated by layers of glass wool (see Figure S7), for controlled heating. Temperatures of the inlet gas and near the sample surface were measured with K-type thermocouples. Feed gas mixtures (O_2_) with Ar as a balance were introduced with mass flow controllers (Bronkhorst Hi-Tech, Low-Δ*P*-flow). The liquid reactant was introduced via a gas saturator with Ar as a carrier gas resulting in adjustable concentrations between 20 and 2000 ppm. The total flow corresponds to 1500 mL_*n*_/min and a weighted hourly space velocity of 1.7 for the catalytic experiment. The outlet of the reactor was connected to the FTIR analyzer and mass spectrometer described above via heated Swagelock tubes and connections.

## Results and Discussion

The expansion of analytical methods and capabilities of commercial analytical instruments is highly desirable as it benefits many R&D activities that need to rely to a lesser extent on nonstandardized methodologies and setups. Here, we present a way forward to speciate and quantify complex HC gas streams with high time resolution using the combination of on-line FTIR and IMR-MS. We use the catalytic conversion of 2,5-dimethylfuran into BTX as a viable case. This reaction may result in a large number of conversion products beside the targeted BTX compounds depending on the catalyst’s selectivity. As an example, the initial GC–MS analysis, where conversion products from a diluted stream were adsorbed onto multisorbent tubes, separated via gas chromatography and analyzed with mass spectrometry, reveals many compounds summarized in Table 1.

**Table 1:** “Heavy Fraction” Molecules during 2,5-dmf Conversion Over a Zeolite (ZSM5-SAR330) Identified with GC–MS Analysis

name	Formula	mass (amu)	toluene equiv (%)
Furans			
2,5-dimethylfuran	C_8_H_8_O	96	75
2,4-dimethylfuran	C_8_H_8_O	96	14
2-methylfuran	C_5_H_6_O	82	0.7
2,3,5-trimethylfuran	C_7_H_10_O	110	0.1
BTX			
benzene	C_6_H_6_	78	6.0
toluene	C_7_H_8_	92	0.9
xylenes	C_8_H_10_	106	0.2
C5-Rings			
2-methyl-2-cyclopenten-1-one	C_6_H_8_O	96	0.7
3-methyl-2-cyclopenten-1-one	C_6_H_8_O	96	0.2
3-methylene-cyclopentene	C_6_H_8_	80	0.2
5-methylcyclopenta-1,3-diene	C_6_H_8_	80	1.0
C7+-Rings and Polycycles			
1,3,5-cycloheptatriene	C_7_H_8_	92	1.0
indene	C_9_H_8_	116	0.2
naphthalene	C_10_H_8_	128	0.1
1-methylnaphthalene	C_11_H_10_	142	0.1
1,5-dimethylnaphthalene	C_12_H_12_	156	0.1

Light gases, for example, ethene, CO, and CO_2_, are excluded such that the collected molecules represent the “heavy” fraction of the product stream. As can be seen, the conversion product stream contains a few isomerization products of the reactant including 2,4-dimethylfuran, 2-methyl-2-cyclopenten-1-one, 3-methyl-2-cyclopenten-1-one, and BTX aromatics and other heavier aromatics such as naphthalene (cf. Figures S9–S14). In addition, the literature provides information on possible products resulting from reactions of furanics over zeolites such as lighter HCs including methane, ethene, and propene and other heavier aromatics such as styrene and indene,^11,15,27^ which in many respects are supported by present GC–MS results.

Using FTIR spectroscopy to analyze the conversion product stream, its rich composition will of course give rise to the corresponding complex infrared spectra with many absorption bands (peaks). Through extensive screening of the sample spectra for pronounced peaks and matching these against the calibration files in the commercial reference compound library, several peaks can be directly assigned to certain compounds. For example, many of the identified compounds such as BTX and small alkenes are part of this library that has been built up over the years by the manufacturer. However, to resolve the full complexity of the FTIR spectra and use all information for compound identification, further in-depth analysis is required. The biobased reactant 2,5-dimethylfuran and some other less-common compounds are not included in this library. Furthermore, IR spectra for 2,4-dimethylfuran and 2-methyl-2-cyclopenten-1-one seem unreported or at least not easily available.

**Figure 1:**
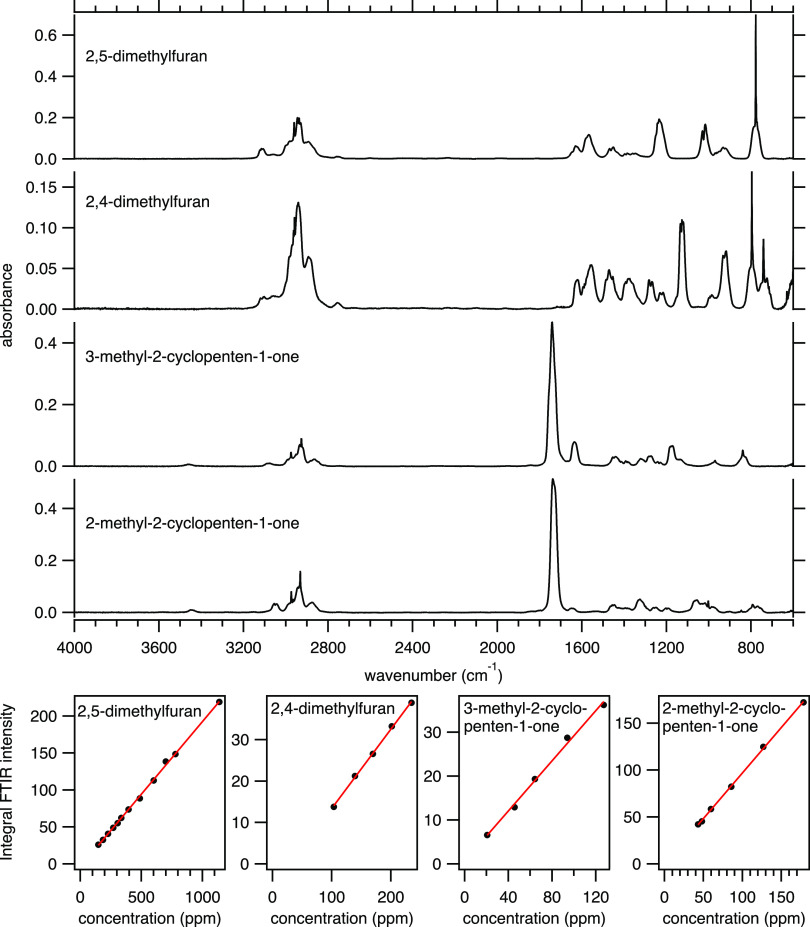
Gas-phase FTIR spectra and fitting of the corresponding FTIR intensity versus the measured concentration of 2,5-dimethylfuran, 2,4-dimethylfuran, 3-methyl-2-cyclopenten-1-one, and 2-methyl-2-cyclopenten-1-one.

To facilitate full use of the spectral information, the commercial library was complemented with in-house calibrations for pure furan, 2-methylfuran, 2,4-dimethylfuran, 2,5-dimethylfuran, 2-methyl-2-cyclopenten-1-one, and 3-methyl-2-cyclopenten-1-one using their full spectral width (mid-IR). The spectra of the latter four are reported in Figure 1. The calibrations were created by introducing known concentrations of the gaseous analyte balanced with argon to the FTIR spectrometer, that is, the use of a series of different concentration results in the corresponding series of absorbance spectra. In Figure 1, the integrated FTIR intensities of the baseline-corrected spectra are plotted against the corresponding concentrations. Good linearity is seen in all cases, which is expected according to the Beer–Lambert law for linear absorbers.

**Figure 2:**
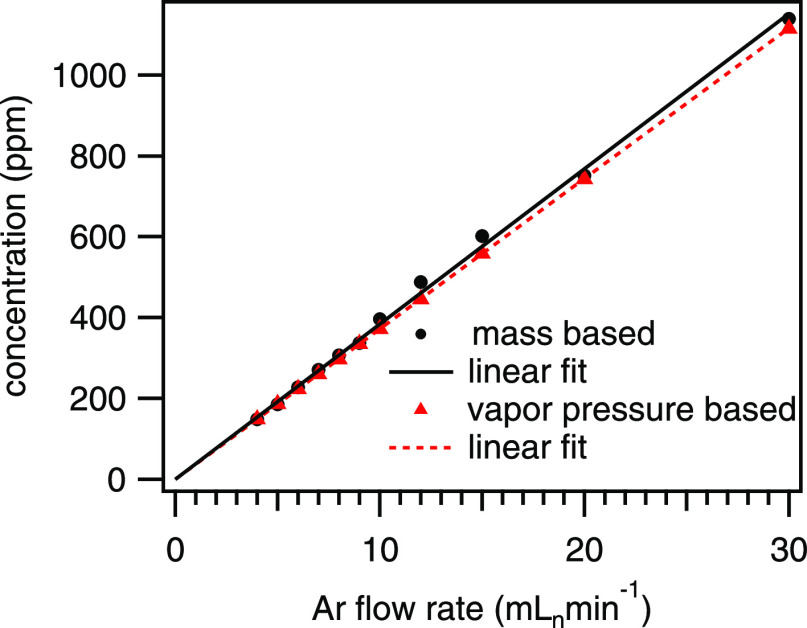
Comparison of the observed mass-based concentration and the calculated vapor pressure-based concentration as a function of the argon flow rate through the gasifier.

We mention that the liquid compounds were transferred into the gas phase by flowing argon carrier gas through a gasifier. The gas-phase concentration is calculated based on the mass difference of the liquid transferred into the carrier stream over a time period of hours. To check the reliability of this approach, the mass-based concentration is compared to the theoretical gas-phase concentration based on the vapor pressure (see Supporting Information) and the flow rate of the carrier gas through the gasifier, as displayed in Figure 2. The minor deviations, caused by an initial overshoot of the mass flow controller feeding the carrier gas through the gasifier, have no significant influence on the targeted accuracy.

**Figure 3:**
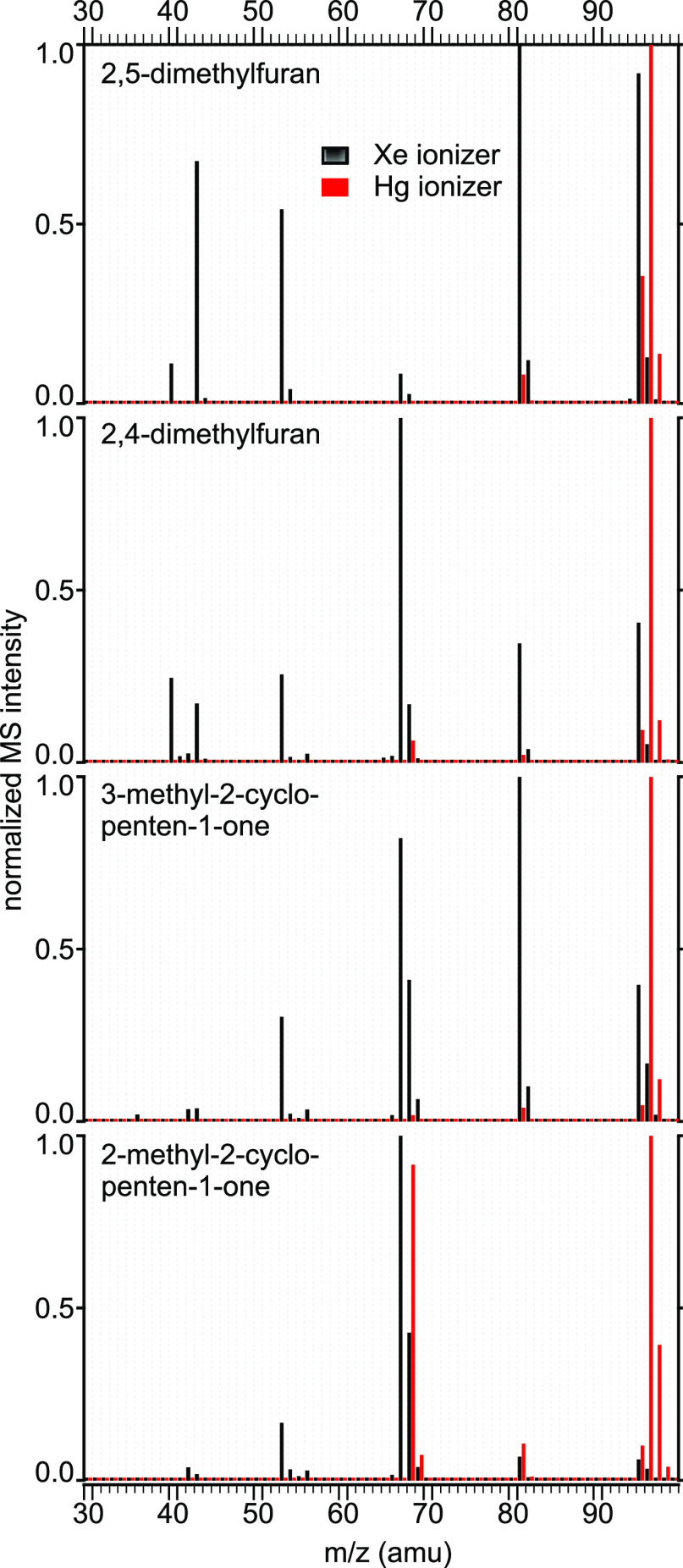
Mass spectra of 2,5-dimethylfuran, 2,4-dimethylfuran, 3-methyl-2-cyclopenten-1-one, and 2-methyl-2-cyclopenten-1-one using Xe (■) and Hg (red ■) as ionizing gases in IMR-MS.

Using mass spectrometry to analyze present conversion products without including a separation step is challenging. A possible approach investigated here is to use IMR-MS for controlled fragmentation. The fragmentation pattern of the analyte is determined by the ionization energy, with fewer fragments generated by collisions with particles of lower energy. The present instrument offers three different ion–molecule reaction pathways thanks to three soft ionizers with different ionization potentials, that is, Hg (10.44 eV), Xe (12.13 eV), and Kr (14.00 eV). As an example, four structural isomers with the same molecular weight are present in the conversion product stream, namely, 2,5-dimethylfuran, 2,4-dimethylfuran, 3-methyl-2-cyclopenten-1-one, and 2-methyl-2-cyclopenten-1-one. The comparison of their MS spectra based on the choice of the ionizers is shown in Figure 3. For each compound, the obtained spectrum shows substantially fewer fragments when using Hg instead of Xe for the IMR ionization. Aside from the parent ion with *m*/*z* = 96, all spectra show a signal at *m*/*z* = 81, which corresponds to the loss of a methyl group. For Hg-IMR, all compounds except 2,5-dimethylfuran show a signal at *m*/*z* = 68. This means that 2,4-dimethylfuran and 2- and 3-methyl-2-cyclopenten-1-one can be distinguished from the reactant 2,5-dimethylfuran since the *m*/*z* = 68 signal is not expected to originate from other compounds identified in the full mixture.

In Table 2 the compounds (molecular formulae) and the conditions (*m*/*z* ratio and wavenumber region) whereby they can be followed by the described methods are summarized. All primary analysis bands have been carefully chosen such that band overlap is minimized and no false-positives are measured.^21,28^ The use of primary analysis bands implicitly means that parts of the reference spectrum for each species are not used for the concentration determination. For a simple mixture without overlapping bands, this may in principle lead to a lower sensitivity, albeit the impact on the quantification is likely negligible. However, for more complex mixtures, as considered here, the concept of primary analysis bands must be used as the impact of overlapping bands is of far more concern than that of limited spectral width. We stress that a few compounds identified by GC–MS are hardly available, such as 1,3,5-cycloheptatriene, or cannot be suitably calibrated with FTIR due to the lack of molecular stability as for 5-methylcyclopenta-1,3-diene. The latter, however, could be tracked via MS based on its specific *m*/*z* ratio of 80 when no cross talking to other analytes is expected.

**Table 2:** Molecules Analyzed Simultaneously in the Complex Gas Stream with Their Chosen *m*/*z* and Primary IR Band

compound	formula	*m*/*z*	IR band (cm^–1^)
Other Rings			
2-methylnaphthalene	C_11_H_10_	142	
naphthalene	C_10_H_8_	128	758.62–807.32
2-methyl-2-cyclopentenone	C_8_H_8_O	(96), 68	1668.88–1809.90
3-methyl-2-cyclopentenone	C_8_H_8_O	(96), 68	1701.42–1811.83
Furans			
2,5-dimethylfuran	C_8_H_8_O	96, (81)	1168.43–1282.69
2,4-dimethylfuran	C_8_H_8_O	(96), 68	1074.17–1174.70
2-methylfuran	C_7_H_6_O	(81)	1117.57–1176.87
BTX			
benzene	C_6_H_6_	78	606.51–726.80
toluene	C_7_H_8_	92	689.44–769.95
*o*-xylene	C_8_H_10_	106	702.45–779.59
*p*-xylene	C_8_H_10_	106	735.32–867.92
Olefins			
ethene	C_2_H_4_	(28), 27	900.12–1000.16
propene	C_3_H_6_	42, (41)	900.61–1019.69
1,3-butadiene	C_4_H_8_	(54), 39	2698.93–2822.36
C1			
methane	CH_4_		3000.25–3176.23
carbon monoxide	CO	28	2146.16–2159.90
carbon dioxide	CO_2_	44	2223.57–2280.94
formaldehyde	CH_2_O	(30)	2698.93–2822.36
water	H_2_O	18	1416.97–1502.31

**Figure 4:**
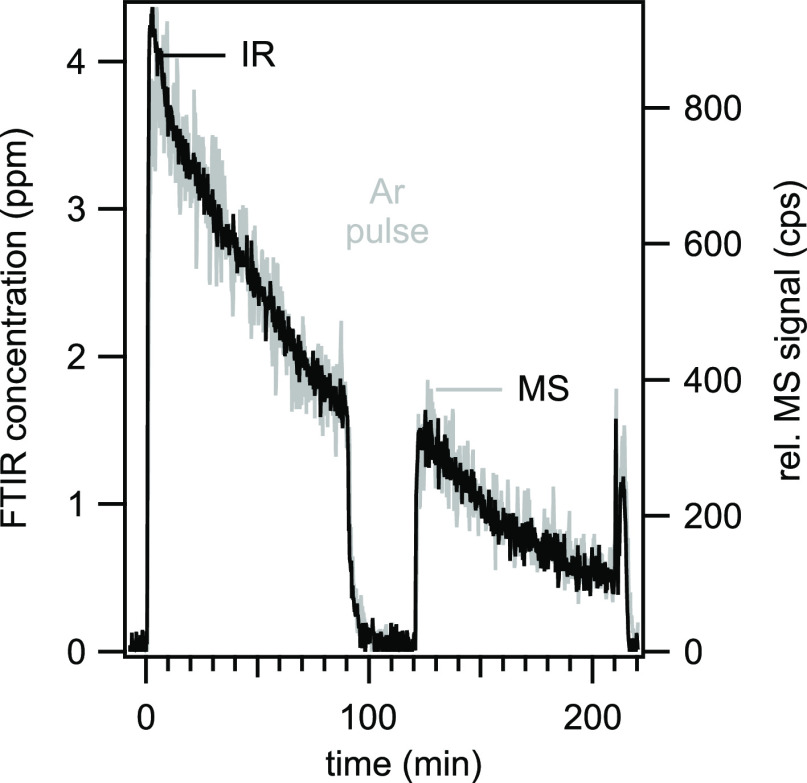
Comparison of the concentration profiles of ethene using FTIR-spectroscopy and mass spectrometry during two 90 min pulses of 2,5-dimethylfuran over zeolite β.

The combined use of FTIR spectroscopy and IMR-MS has the advantage that one method can be used to support the other for certain compounds. As an example, ethene was tracked by IMR-MS using Hg as an ionizer for a clear signal at *m*/*z* 27 and used to check the reliability of the FTIR signal. The two measured signals are compared in Figure 4. As can be seen, the correspondence between the two is high, which confirms the agreement in this case. A similar consistency is observed also for propene and toluene (see Figures S9–S14 in Supporting Information for the concentration profiles of all analyzed species). For some other compounds, the concentrations may be too low to be accurately followed by FTIR. This is the case for xylenes in our study. Fortunately, xylenes can be qualitatively measured with IMR-MS on *m*/*z* 106 with the only drawback that different xylenes cannot be distinguished well.

**Figure 5:**
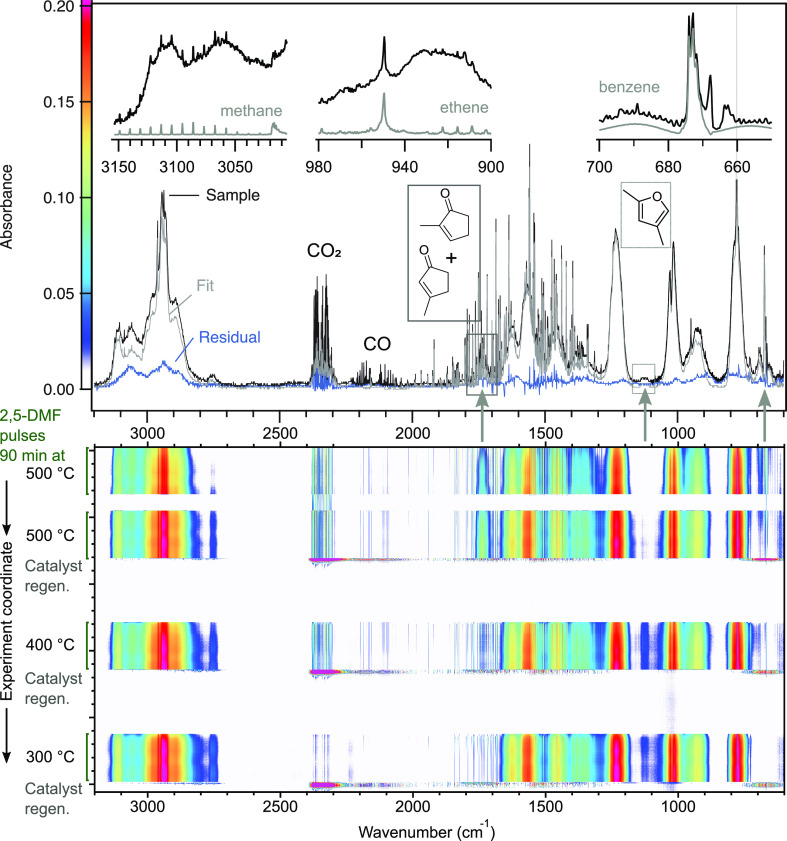
Top: FTIR spectra of the sample gas composition, the fitting based on the analyzed species, and the residual during 2,5-dimethylfuran conversion over zeolite β at 500 °C and highlighted peak selections and their attributions. Bottom: Contour plot of the FTIR signal intensity during the whole experiment (four 2,5-dmf pulses at 500, 400, and 300 °C and oxidative regeneration of the catalyst).

With the abovementioned information at hand, it is now possible to perform compositional analyses of the complex HC streams. Figure 5 presents spectroscopic data for the product stream from a real catalytic experiment, namely, the conversion of 2,5-dmf over zeolite β at 500 °C. The top panel shows a complete sample spectrum of the full product stream at the initial conversion over zeolite β at 500 °C together with the fitted and residual spectra. The reactant 2,5-dmf has the strongest contribution to the sample spectrum because the experiment is carried out under differential (low conversion) operation conditions according to the established practice of catalyst evaluation. The top panel also shows the characteristic absorbance bands for the identified species methane, ethene, and benzene. Furthermore, CO_2_, CO, 2,4-dimethylfuran, and 2- and 3-methyl-2-cyclopenten-1-one are highlighted. In the bottom panel, the intensity of the different absorbance bands is visualized as a 2D contour map. In the experiment, the catalyst is exposed to 2,5-dimethylfuran for 90 min reaction periods at 500, 400, and 300 °C (for the experimental sequence, also see Figure S8). An increase in peak intensities is clearly observed for the pronounced C=O stretching band representing the carbonyls of 2- and 3-methyl-2-cyclopenten-1-one, whereas the benzene band around 675 cm^–1^ is decreasing with time on stream.

**Figure 6:**
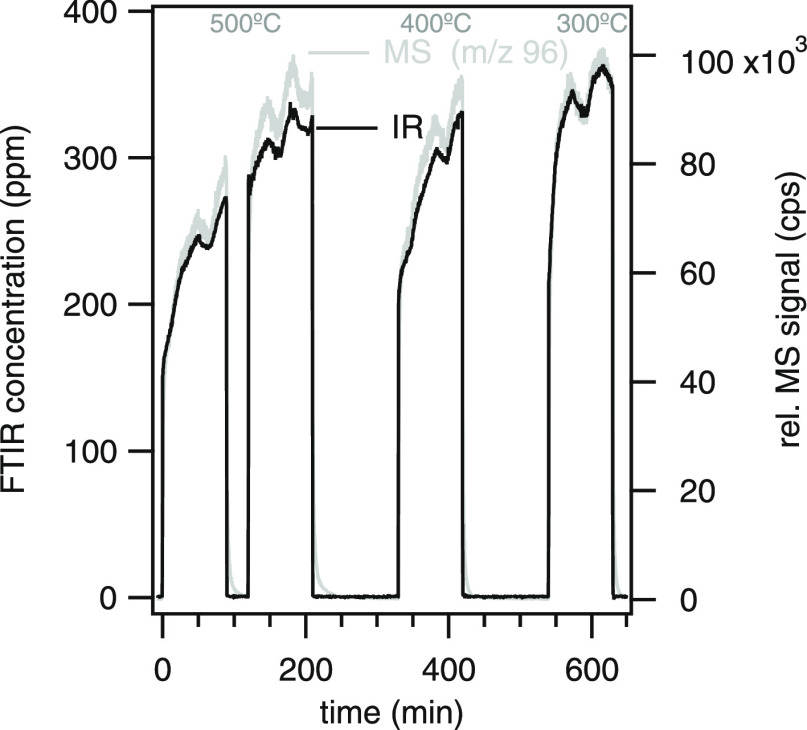
Concentration profile during the whole catalytic experiment at 500, 400, and 300 °C for 2,5-dimethylfuran measured by IR and its corresponding MS signal for *m*/*z* = 96. Because 2,4-dimethylfuran and 2- and 3-methyl-cyclopenten-1-one are all measured at *m*/*z* = 96, the IR and MS signals differ at 500 and 400 °C when those product concentrations are relatively high.

This trend of decreasing selectivity toward olefins and aromatics, while isomerization products are increasing, is also visible in the concentration profiles of the specific compounds (cf. Supporting Information). As one example, the complete concentration profile of 2,5-dmf during the whole catalytic experiment is presented in Figure 6. As can be seen, the IR and MS signal differ at 500 and 400 °C compared to that at 300 °C. This can be explained by the fact that besides 2,5-dmf, its isomers 2,4-dimethylfuran and 2- and 3-methyl-cyclopenten-1-one are measured at *m*/*z* = 96. At the two higher temperatures, the production of the isomers is increased resulting in a stronger MS signal. The changes in selectivity are related to the buildup of coke and carbonaceous species on the catalyst’s surface. Although a deeper analysis of underlying catalytic phenomena is beyond the scope of the present study, we will point out a few more advantages with the on-line methodology.

**Figure 7:**
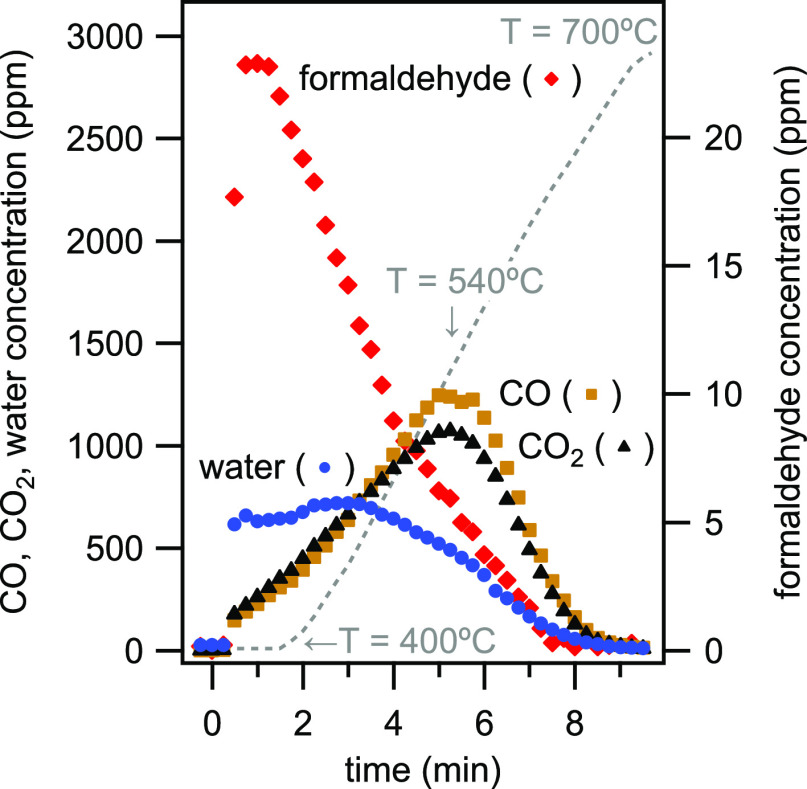
Concentration profiles of oxidation products CO, CO_2_, water, and formaldehyde during the oxidative catalyst regeneration (20% O_2_) and heat ramp 400 to 700 °C after 2,5-dimethylfuran conversion at 400 °C.

After each reaction period, the catalyst was treated with 20% O_2_ during a heating ramp from the reaction temperature up to 700 °C to remove coke and carbonaceous species from the catalyst surface. The on-line method allows for in situ characterization of the effluent composition during this regeneration process. As can be seen in Figure 7, water, CO, CO_2_, and formaldehyde all form during the catalyst regeneration. Water and formaldehyde form at lower temperatures, whereas the formations of CO and CO_2_ occur at higher temperatures with maxima at around 540 °C. The formation maxima of CO and CO_2_ align rather well with the noncatalytic oxidation of soot formed from oxygenates.^29^ In addition to the in situ speciation, integration and summation of the different traces reveal the amount of carbon deposited on the catalyst during the reaction period. In this way, all information to calculate a carbon balance is directly available without the need of collecting (trapping) CO, CO_2_, and formaldehyde to measure their weight, which facilitates studies of catalyst deactivation.^30,31^

Here, the carbon balance closes at 89, 93, and 97% at 500, 400, and 300 °C, respectively. This is comparable to GC–MS analyses of similar CFP processes.^10^ The lower percentage for higher temperatures is readily explained by the lower catalyst selectivity, that is, more reaction pathways become possible with increased temperature, resulting in side products that are not included in the calibration.

**Figure 8:**
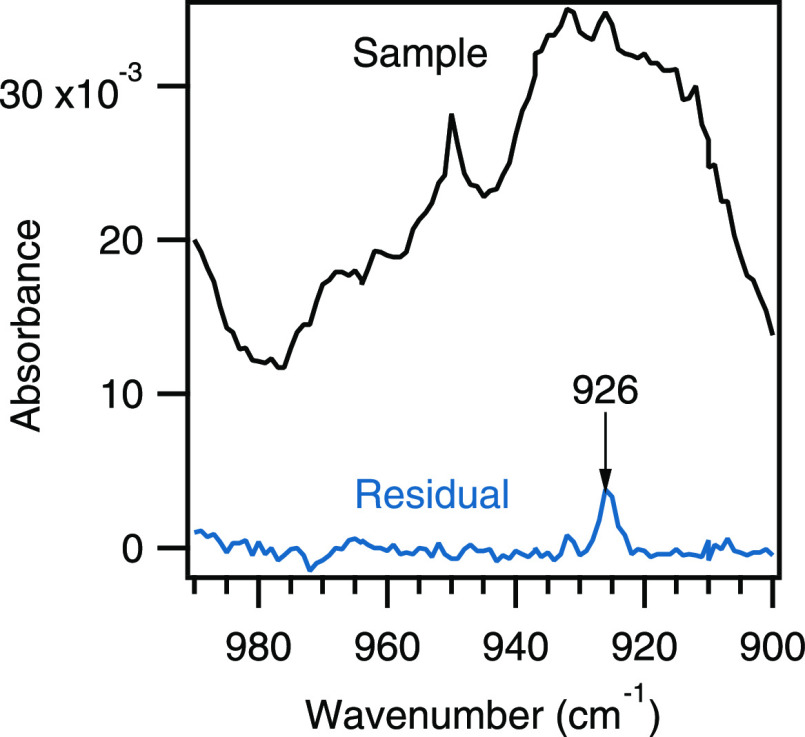
Infrared spectrum of the sample and the residual showing an unidentified species with a band at 926 cm^–1^.

In summary, the presented methodology exhibits both advantages and disadvantages. A limitation is that only species that have been identified as part of the stream, accurately calibrated and added to the reference compound library, can be quantified in the on-line FTIR mode. This means that under significantly different conditions, for example, in the case of another feedstock and/or a different catalyst for which the formation of other products can be expected, new calibrations need to be carried out if these products are of interest to monitor. Furthermore, the choice of primary analysis bands requires caution to avoid cross talking between analytes and to avoid overestimation of their concentrations, especially in the case of another species that is an absorber in the same wavenumber region as an analyte of interest but not part of the reference compound library. However, the presence of an unknown species does not necessarily need to influence the measurement of the species of interest, although it cannot a priori be ruled out. The most straightforward way to judge this is most likely to analyze the goodness of fit alongside the residual spectra, as shown above, because the creation of a well-defined complex gas mixture out of a liquid mixture to be used for validation is not an easy task. Again, we point out that signatures different from random noise in the residual spectra reveal that they are not part of the fitting procedure, that is, not contributing to determined concentrations, but indicate unidentified species, such as the band at 926 cm^–1^ presented in Figure 8.

Concerning the IMR-MS part, a limitation is that despite the use of soft ionization, the signal for benzene is influenced by the presence of toluene and xylenes (cross talking).

The mentioned limitations are by no means unique but rather common for many analysis methods. In fact, the presented method has more advantages. First, it makes on-line quantification possible. Second, it allows for automation opportunities. As an example, catalyst evaluation often relies on exposing the catalyst to systematically varied reaction conditions (feed composition and temperature), which may include pre- and post-treatment steps and intermittent regeneration sequences, while characterizing the conversion product stream on-line. With the present method, all these steps can be evaluated using the same chemical reactor system and catalyst. This is of high value when studying powder samples that are difficult to transfer between different experimental equipment without changing or losing the catalyst material. Third, it may serve or be adapted for on-line chemical product monitoring and/or chemical process control, which is one of the principles of green chemistry.

## Conclusions

This work shows that FTIR can be combined with MS to realize a method for on-line composition analysis of a simulated processed plant biomass stream. The conversion of 2,5-dimethylfuran over a zeolite has been chosen as a model system because it offers the prospect to selectively produce valuable green BTX aromatics and olefins. It is possible to track the concentrations of a multitude of conversion products simultaneously without a separation step and with a time resolution in the order of seconds. The opportunity to calculate a carbon balance exists by in situ oxidative regeneration of the catalyst. It is shown that most conversion species are identified by a carbon balance of ca. 90% and that their concentrations are quantified in the on-line method. This approach allows further for high automation of experimental analysis in heterogeneous catalysis and offers the potential for on-line production monitoring.

## Supporting Information Available

Chemicals; materials; spectral data: gas-phase infrared spectra for 2,5-dimethylfuran, 2,4-dimethylfuran, 2-methyl-cyclopenten-2-one, and 3-methyl-cyclopenten-2-one; ^1^H- and ^13^C NMR spectra of 2,4-dimethylfuran; schematic description of the chemical flow reactor; description of the experimental sequence during the catalytic experiment; details about the vapor pressure-based concentration calculation; and concentration profiles of the analyzed conversion products during the catalytic experiment (PDF)
