# Supplementary figures and images for: On-Line Composition Analysis of Complex Hydrocarbon Streams by Time-Resolved Fourier Transform Infrared Spectroscopy and Ion–Molecule Reaction Mass Spectrometry
Source: Anal Chem. 2021 Sep 22;93(39):13187–95. doi: 10.1021/acs.analchem.1c01929 (PMC8495676; doi:10.1021/acs.analchem.1c01929)

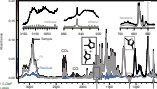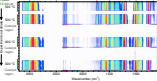

Supplement: Supplementary file 1 — ac1c01929_si_001.pdf [file ac1c01929_si_001.pdf]
